# The incidence and survival of pancreatic cancer by histology, including rare subtypes: a nation‐wide cancer registry‐based study from Taiwan

**DOI:** 10.1002/cam4.1795

**Published:** 2018-09-27

**Authors:** Jeffrey S. Chang, Li‐Tzong Chen, Yan‐Shen Shan, Pei‐Yi Chu, Chia‐Rung Tsai, Hui‐Jen Tsai

**Affiliations:** ^1^ National Institute of Cancer Research National Health Research Institutes Tainan Taiwan; ^2^ Department of Internal Medicine National Cheng Kung University Hospital National Cheng Kung University Tainan Taiwan; ^3^ Department of Internal Medicine Kaohsiung Medical University Hospital, Kaohsiung Medical University Kaohsiung Taiwan; ^4^ Institute of Molecular Medicine National Cheng Kung University Tainan Taiwan; ^5^ Department of Surgery National Cheng Kung University Hospital Tainan Taiwan; ^6^ Institute of Clinical Medicine National Cheng Kung University Tainan Taiwan; ^7^ Department of Pathology Show Chwan Memorial Hospital Changhua Taiwan; ^8^ School of Medicine College of Medicine Fu Jen Catholic University New Taipei City Taiwan

**Keywords:** incidence, pancreatic cancer, survival

## Abstract

Studies have indicated a significant rise in the incidence of pancreatic adenocarcinoma. However, the epidemiology of other rare histologic subtypes of pancreatic cancer is not well understood. This study analyzed the incidence and survival of pancreatic cancer in Taiwan by histologic subtype, sex, age group, and year of diagnosis. The incidence trends of pancreatic cancer in Taiwan from 2002 to 2013 were calculated using data from the Taiwan Cancer Registry. The survival of pancreatic cancer patients was assessed using the life‐table method and Cox proportional hazards analysis. The incidence of pancreatic cancer increased from 4.62 per 100,000 in 2002 to 6.04 per 100,000 in 2013 in Taiwan. The most common histologic subtype of pancreatic cancer was adenocarcinoma followed by carcinoma and neuroendocrine tumors (NETs). Adenocarcinoma and NETs showed a rapid increase in incidence, while the incidences of other subtypes did not change significantly. Patients with adenocarcinoma showed a poor survival with a 5‐year survival of 5.2%. Patients with endocrinomas, NETs, and lymphoma displayed a better survival than those with adenocarcinoma, with a 5‐year survival ranging from 41.8% to 59.1%. The survival of adenocarcinoma, lymphoma, and NETs improved after the introduction of novel therapies. Understanding the risk factors and identifying the biomarkers for the early diagnosis of pancreatic cancer are important to prevent the development and improve the survival of pancreatic cancer.

## 1 INTRODUCTION

Pancreatic cancer is a cancer with high mortality. The incidence of pancreatic cancer varies across different regions with higher incidence rates in Northern America (7.4 per 100 000) and Western Europe (7.3 per 100 000) and lower incidence rates in Middle Africa and South Central Asia (approximately 1.0 per 100 000).^1^ The mortality of pancreatic cancer also varies across different regions. Higher mortality rates were reported in Northern America (6.9 per 100 000) and Western Europe (6.8 per 100 000), and lower mortality rates were reported in Middle African and South Central Asian countries (<1 per 100,000).^1^ The incidence and mortality rates of pancreatic cancer are also high in Taiwan. In Taiwan in 2013, 2051 individuals were diagnosed with pancreatic cancer (age‐standardized incidence rate = 6.0 per 100 000), and 1798 individuals died of pancreatic cancer (age‐standardized mortality rate = 5.22 per 100 000). Pancreatic cancer accounted for 2.07% of the total cancer incidence and 4.10% of all‐cancer mortality in Taiwan in 2013.^2^

High incidence and mortality rates of pancreatic cancer in other Asian countries, including Korea and China, have also been reported. Jung et al. reported that the incidence rate of pancreatic cancer increased from 5.6 per 100 000 in 1999 to 6.1 per 100 000 in 2007 in Korea according to the Korea Central Cancer Registry (KCCR), a nation‐wide hospital‐based cancer registry. The mortality rate of pancreatic cancer in Korea was 5.5 per 100 000 in 2006 and 2007.^3^ Chen et al. reported a pancreatic cancer incidence rate of 4.63 per 100 000 and a mortality rate of 4.15 per 100 000 in 2009 according to the National Central Cancer Registry of China.^4^ However, another report from China by Luo et al. showed that the incidence rate of pancreatic cancer was approximately 6.7 per 100 000 from 2004 to 2009 according to the Shanghai Cancer Registry. In addition, they reported that the 1‐year, 3‐year, and 5‐year survival rates of pancreatic cancer were 17.8%, 5.7%, and 4.1%, respectively.^5^ Egawa et al. also reported a low survival rate of pancreatic cancers in Japan.^6^ The 5‐year survival rate from 2001 to 2007 was 18.8% for resectable tumors and only 3.1% for unresectable tumors.^6^ The incidence and mortality rate of Hong Kong, where is located near Taiwan, were 4.1 and 3.7 per 100 000, respectively, in 2012 according to Hong Kong Cancer Registry.^7^ According to the International Agency for Research on Cancer (IARC), the estimated incidence rate in Asia was variable among different countries in 2012.^8^ The highest incidence rates were 8.5 and 6.7 per 100 000 in Japan and Korea, respectively. The mortality was also high with 7.7 and 6.2 per 100 000 in Japan and Korea, respectively. By contrast, a lower incidence rate was noted in some countries, such as India, Vietnam, and Bangladesh with 1.2, 1, and 0.7 per 100 000, respectively.^8^Table S1 shows the estimated incidence and mortality rates of pancreatic cancer in some Asian countries according to IARC and the incidence and mortality in Hong Kong and Taiwan according to the cancer registry in Hong Kong and Taiwan.^2^, ^7^, ^8^

The poor survival of pancreatic cancer is universal worldwide with the mortality rate almost equaling the incidence rate. The overall 5‐year survival rate is approximately 6% (2%‐9%).^1^ Most pancreatic cancer cases are adenocarcinoma; thus, most cancer registries or studies of pancreatic cancer are focused on adenocarcinoma. However, in addition to adenocarcinoma, there are other histologic subtypes, including neuroendocrine tumors (NETs), small cell carcinoma, sarcoma, and lymphoma. These subtypes are quite different from pancreatic adenocarcinoma. Among them, NETs are the second most common subtype of pancreatic cancer with a relatively longer survival. Although adenocarcinoma has been well studied, data on the incidence and survival of the other histologic subtypes of pancreatic cancer have been more limited. In addition, a nation‐wide population‐based study for pancreatic cancer, including rare subtypes, is lacking in Asia. Therefore, we used the Taiwan Cancer Registry (TCR) database to analyze the incidence, distribution, and survival of pancreatic cancer in Taiwan. The aim of this study was to comprehensively evaluate the trends in the incidence, distribution, and survival of different subtypes of pancreatic cancer in Taiwan.

## 2 MATERIALS AND METHODS

This study was approved by the Research Ethics Committee of the National Health Research Institutes, Taiwan. Because this study used de‐identified secondary data, no individual consent was required.

The data used for the current analysis were obtained from the TCR and Death Registry Database housed in the Health and Welfare Data Science Center, Ministry of Health and Welfare, Taiwan. The TCR, established in 1979 to monitor the incidence and mortality rates of cancer in Taiwan, includes approximately 97% of the cancer cases occurring in Taiwan.

The incident cases of pancreatic cancer diagnosed in Taiwan between 1 January 2002 and 31 December 2013, were identified from the TCR using the topography codes of the International Classification of Diseases for Oncology, Third Edition (ICD‐O‐3). The histologic subtypes of pancreatic cancer were assigned according to the morphology (M) codes and included adenocarcinoma, carcinoma, NETs, endocrinomas, lymphoma, squamous cell carcinoma, small cell carcinoma, and sarcoma. The M codes for NETs were defined previously.^9^ Endocrinomas are functional tumors coded as 8150‐8157.

The annual populations reported by the Directorate‐General of Budget, Accounting, and Statistics of Taiwan (http://www.dgbas.gov.tw) were used as denominators to calculate the crude annual incidence rates of pancreatic cancer in Taiwan from 2002 to 2013 by histologic subtype, sex, and age group. The crude rates were then age‐standardized to the 2000 WHO standard population to generate the age‐standardized incidence rates. The annual percentage change (APC) was estimated to evaluate the incidence trends of pancreatic cancer overall and by histologic subtype using linear regression: log (rate_y_) = b_0_ + b_1_y, with log(rate_y_) = natural log of incidence rate in year y. APC = (e^b1^‐1)×100. A *P* < 0.05 indicated a significant change in the incidence trend.

The data on vital status and the date of death were ascertained from the Death Registry Database. One‐, three‐, five‐, and ten‐year survival rates of pancreatic cancer overall, by sex, histologic subtype, and time period of diagnosis were calculated using the life‐table method. The hazard ratio (HR) and 95% confidence interval (CI) of pancreatic cancer death associated with the histologic subtype, age, sex, and time period of diagnosis were estimated using the Cox proportional hazards survival analysis. Survival analysis was first performed using pancreatic cancer diagnosed during 2002‐2013, and using two time periods, 2002‐2007 (T1) and 2008‐2013 (T2).

## 3 RESULTS

### 3.1 Age‐standardized incidence rates

**Figure 1:**
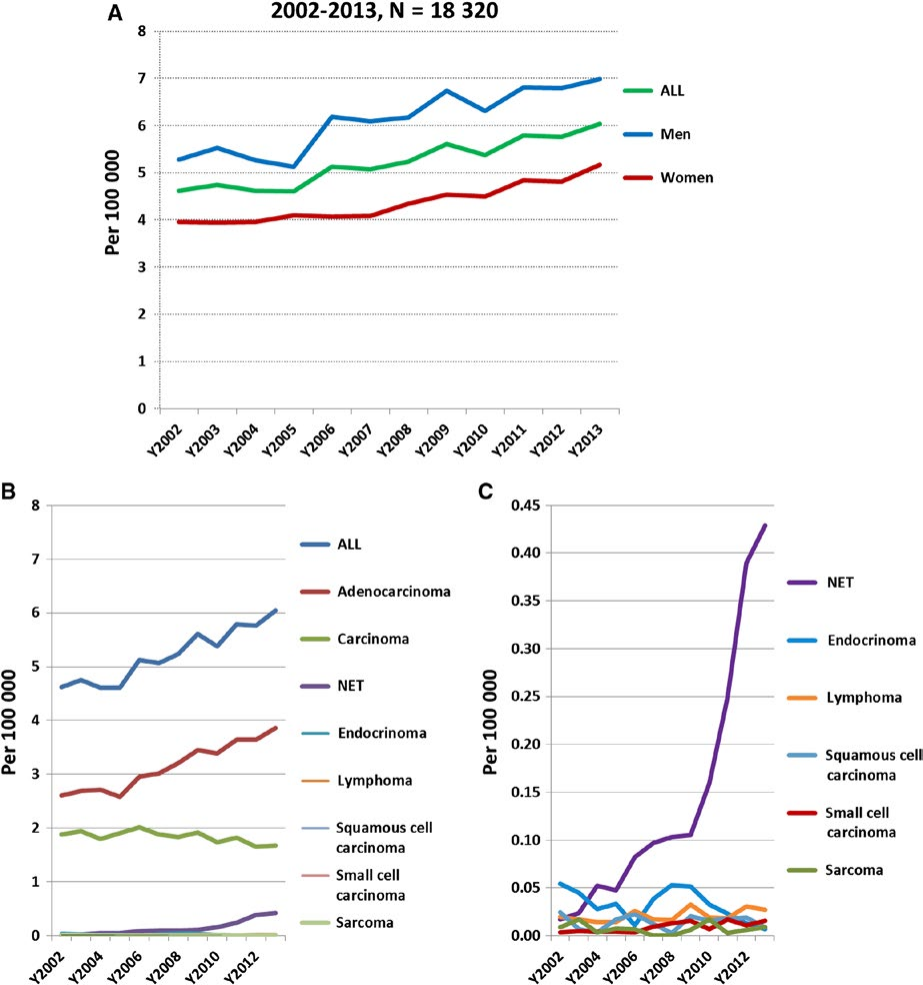
The annual age‐standardized incidence rate (cases per 100 000) of pancreatic cancers from 2002 to 2013 in Taiwan. (A) The annual age‐standardized incidence rate of pancreatic cancers in men, women, and both sexes from 2002 to 2013 in Taiwan. (B) The annual incidence rate of pancreatic cancer by histologic subtype. (C) The annual incidence rate of the rare histologic subtype of pancreatic cancer, including neuroendocrine tumors (NETs), endocrinomas, squamous cell carcinoma, lymphoma, small cell carcinoma, and sarcoma

In total, 18 320 newly diagnosed pancreatic cancer cases were recorded in the TCR from 1 January 2002 to 31 December 2013, comprising 10 520 (57.4%) men and 7800 (42.6%) women. The mean age was 67.3 years for all subjects—66.7 years for men and 68.1 years for women. The age‐standardized incidence rate of pancreatic cancer in Taiwan increased from 4.62 per 100 000 in 2002 to 6.04 per 100 000 in 2013 (APC = 2.6, *P *= 1 × 10^−6^) (Figure 1A and Table S2). Men displayed a higher incidence rate of pancreatic cancer than women during this time period. For men, the incidence rate of pancreatic cancer was 5.29 per 100 000 in 2002 and increased to 6.99 per 100 000 in 2013 (APC = 2.85, *P *= 0.00004). For women, the incidence rate of pancreatic cancer increased from 3.96 per 100 000 in 2002 to 5.16 per 100 000 in 2013 (APC = 2.46, *P *= 1×10^−6^). The increasing trend was similar for both men and women.

The incidence rates of all subtypes of pancreatic cancers are presented in Figure 1B and Table S2. The histologic subtypes were classified as adenocarcinoma, NETs (neuroendocrine carcinoma included), endocrinomas, lymphoma, squamous cell carcinoma, small cell carcinoma, and sarcoma. In addition, some cases were classified as carcinoma because they were coded as carcinoma but not classified as adenocarcinoma or other type of carcinoma in the TCR database. The incidence rate of adenocarcinoma increased from 2.61 per 100 000 in 2002 to 3.87 per 100 000 in 2013 (APC = 3.94, *P *= 2×10^−7^). The incidence rate of carcinoma was 1.89 per 100 000 in 2002, increased to 2.01 per 100 000 in 2006, and then decreased gradually to 1.68 per 100 000 in 2013. The incidence rate of NETs increased from 0.02 per 100 000 in 2002 to 0.43 per 100 000 in 2013 with an APC of 31.52 (*P *= 3×10^−8^). The increased incidence was observed in both men and women. The incidence rate of NETs in men increased from 0.02 per 100 000 in 2002 to 0.41 per 100 000 in 2013 with an APC of 34.02 (*P *= 3×10^−8^). The incidence rate of NETs in women was 0.02 per 100 000 in 2002 and became 0.45 per 100 000 in 2013 with an APC of 32.48 (*P *= 0.00004). The incidence rate of small cell carcinoma was 0.004 per 100 000 in 2002 and became 0.02 per 100 000 in 2013 (APC = 15.23, *P* = 0.002). The annual incidence rates of endocrinomas, lymphoma, squamous cell carcinoma, and sarcoma did not change significantly. The annual incidence rates of the rare subtypes of pancreatic cancers, including NETs, endocrinomas, squamous cell carcinoma, small cell carcinoma, and sarcoma, are further highlighted in Figure 1C.

### 3.2 Distribution and incidence trends of pancreatic cancer by age, sex, and subtype

**Figure 2:**
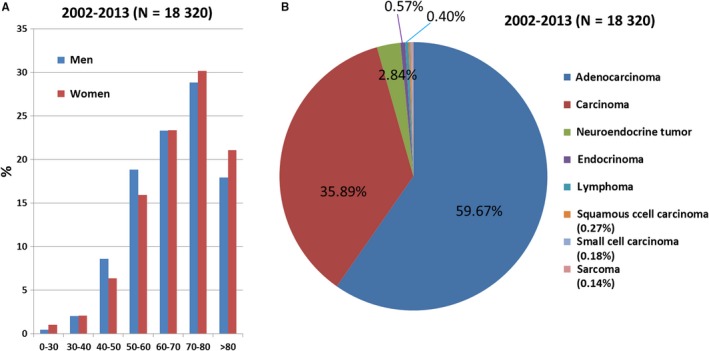
The distribution of pancreatic cancers by age, sex, and histologic subtype. (A) The proportion of pancreatic cancers by age and sex from 2002 to 2013. (B) The distribution of pancreatic cancers by histologic subtypes from 2002 to 2013

The distribution of pancreatic cancer by age group and sex is presented in Figure 2A. Those aged between 70 and 80 years accounted for the largest proportion (29.4%), and 72.0% of pancreatic cancers were diagnosed at ≥60 years of age. The age distribution was not significantly different between men and women. We analyzed the distribution of pancreatic cancer by histologic subtype from 2002 to 2013 (Figure 2B). Adenocarcinoma (59.67%) was the most common histologic subtype of pancreatic cancer, followed by carcinoma (35.89%), NETs (2.84%), endocrinomas (0.57%), lymphoma (0.40%), squamous cell carcinoma (0.27%), small cell carcinoma (0.18%), and sarcoma (0.14%).

The distribution of pancreatic cancer by subtype, sex, and age group was evaluated using two time periods, 2002‐2007 (T1) and 2008‐2013 (T2), as shown in Table 1. The most common age of diagnosis was between 70 and 80 years for both time periods (32.22% in T1 and 27.44% in T2). The second most common age of diagnosis was between 60 and 70 years, accounting for 23.4% in T1 and 23.27% in T2. The proportion of female pancreatic cancer cases increased slightly over time although without statistical significance (chi‐squared test; *P *= 0.07) and accounted for 41.78% and 43.13% of all pancreatic cancers in T1 and T2, respectively.

**Table 1:** The distributions of pancreatic cancer by age groups, sex, and histologic subtype in two time periods

	2002‐2007 (T1)					2008‐2013 (T2)			*P* [Link] for comparing the distributions between T1 and T2	*P* [Link] for comparing the distributions among men between T1 and T2	*P* [Link] for comparing the distributions among women between T1 and T2
	ALL	Men		Women		ALL	Men	Women			
	N (%)	N (%)		N (%)		N (%)	N (%)	N (%)			
Age											
0‐30	46 (0.61)	21 (0.48)		25 (0.79)		78 (0.72)	24 (0.39)	54 (1.16)	9 × 10^−19^	4 × 10^−16^	0.00001
30‐40	162 (2.15)	92 (2.09)		70 (2.22)		215 (2.0)	123 (2.01)	92 (1.98)			
40‐50	622 (8.24)	397 (9.04)		225 (7.14)		777 (7.21)	506 (8.26)	271 (5.83)			
50‐60	1273 (16.87)	777 (17.69)		496 (15.74)		1954 (18.13)	1206 (19.68)	748 (16.09)			
60‐70	1765 (23.4)	992 (22.59)		773 (24.52)		2508 (23.27)	1460 (23.83)	1048 (22.55)			
70‐80	2431 (32.22)	1445 (32.9)		986 (31.28)		2957 (27.44)	1588 (25.91)	1369 (29.45)			
>80	1245 (16.5)	668 (15.21)		577 (18.31)		2287 (21.22)	1221 (19.92)	1066 (22.93)			
Sex											
Men	4392 (58.22)					6128 (56.87)			0.07		
Women	3152 (41.78)					4648 (43.13)					
Histologic subtype											
Adenocarcinoma	4303 (57.04)		2515 (57.26)		1788 (56.73)	6634 (61.56)	3847 (62.78)	2787 (59.96)	<1 × 10^−30^	9 × 10^−29^	1 × 10^−15^
Carcinoma	3034 (40.22)		1774 (40.39)		1260 (39.97)	3544 (32.89)	1967 (32.1)	1577 (33.93)			
NETs	84 (1.11)		39 (0.89)		45 (1.43)	436 (4.05)	219 (3.57)	217 (4.67)			
Endocrinomas	53 (0.7)		25 (0.57)		28 (0.89)	51 (0.47)	21 (0.34)	30 (0.65)			
Lymphoma	29 (0.38)		16 (0.36)		13 (0.41)	45 (0.42)	27 (0.44)	18 (0.39)			
Squamous cell carcinoma	22 (0.29)		12 (0.27)		10 (0.32)	27 (0.25)	20 (0.33)	7 (0.15)			
Small cell carcinoma	8 (0.11)		4 (0.09)		4 (0.13)	25 (0.23)	20 (0.33)	5 (0.11)			
Sarcoma	11 (0.15)		7 (0.16)		4 (0.13)	14 (0.13)	7 (0.11)	7 (0.15)			

*P* value was calculated by chi‐square test.

The distribution of pancreatic cancers by subtype is shown in Table 1. The distribution of subtypes was significantly different between T1 and T2 for all, or in men or women (chi‐squared test; *P *< 1×10^−30^ for all, *P *= 9×10^−29^ for men and *P *= 1×10^−15^ for women). Adenocarcinoma was the most common subtype of pancreatic cancer for both men and women in both time periods. The percentage of adenocarcinoma increased from 57.04% in T1 to 61.56% in T2. The percentage of carcinoma was 40.22% in T1. However, the percentage of carcinoma was 32.89% in T2. The decrease in the percentage of carcinoma suggested that more cases of carcinoma were classified into the other subtypes of pancreatic cancer in T2. The distribution of NETs was different in the two time periods for both men (0.89% in T1 and 3.57% in T2) and women (1.43% in T1 and 4.67% in T2). The percentage of NETs increased from T1 to T2 for both men and women. For endocrinomas, the percentage decreased slightly over time for both men (0.57% in T1 and 0.34% in T2) and women (0.89% in T1 and 0.65% in T2).

### 3.3 Survival

**Figure 3:**
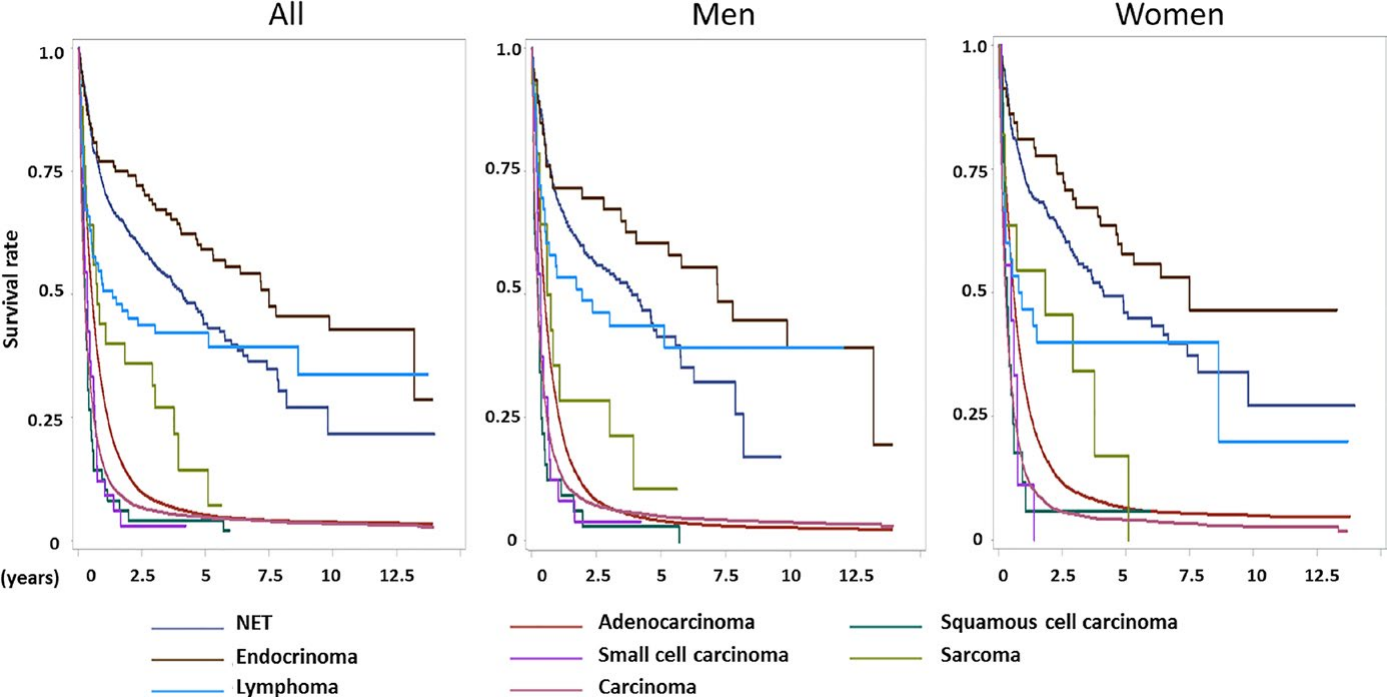
The survival curves of pancreatic cancers by histologic subtypes in all, men and women diagnosed from 2002 to 2013 in Taiwan

The 1‐, 3‐, 5‐, and 10‐year survival rates of pancreatic cancer by subtype and sex are presented in Table 2. The 1‐year, 3‐year, 5‐year, and 10‐year survival rates for all pancreatic cancer patients diagnosed between 2002 and 2013 were 25.52%, 9.22%, 6.6%, and 4.71%, respectively. The 1‐year, 3‐year, 5‐year, and 10‐year survival rates were 24.17%, 8.43%, 5.95%, and 4.24% for men, respectively, and 27.34%, 10.28%, 7.47%, and 5.34% for women, respectively. The survival rates by histologic subtype for both sexes combined and for each sex separately are shown in Figure 3 and Table 2. Among the histologic subtypes, the best survival was seen in patients with endocrinomas, followed by NETs, lymphoma, and sarcoma. However, the long‐term survival (>5 years) was better for lymphoma than NETs as shown in Figure 3. For women, the best survival rate was observed in endocrinomas, followed by NETs and sarcoma and lymphoma. The best survival rate for men was endocrinomas, followed by NETs and lymphoma. The long‐term survival rate (> 5 years) was better for lymphoma than NETs in men as shown in Figure 3.

**Table 2:** 1‐, 3‐, 5‐, and 10‐year survival rates of pancreatic cancers from 2002 to 2013

	1‐year survival rate			3‐year survival rate			5‐year survival rate			10‐year survival rate		
	All	Men	Women	All	Men	Women	All	Men	Women	All	Men	Women
All	0.2552	0.2417	0.2734	0.0922	0.0843	0.1028	0.066	0.0595	0.0747	0.0471	0.0424	0.0534
Adenocarcinoma	0.290	0.2692	0.3181	0.081	0.0692	0.0976	0.052	0.043	0.0651	0.0375	0.0289	0.0496
Carcinoma	0.151	0.1556	0.145	0.0609	0.067	0.0528	0.0471	0.0516	0.0412	0.0344	0.0401	0.027
NETs	0.717	0.6938	0.7405	0.561	0.5464	0.5754	0.445	0.419	0.4694	0.2349	0.1794	0.2796
Endocrinomas	0.769	0.7174	0.8103	0.682	0.6736	0.6897	0.591	0.6054	0.5804	0.431	0.3892	0.4683
Lymphoma	0.507	0.5349	0.4667	0.437	0.4633	0.4	0.418	0.4324	0.4	0.3344	0.3892	0.24
Squamous cell carcinoma	0.122	0.125	0.1176	0.041	0.0313	0.0588	0.041	0.0313	0.0588	‐	‐	‐
Small cell carcinoma	0.121	0.125	0.1111	0.030	0.0417	‐	0.030	0.0417	‐	‐	‐	‐
Sarcoma	0.440	0.3571	0.5455	0.318	0.2857	0.3535	0.159	0.1224	0.2121	‐	‐	‐

We performed Cox proportional hazards survival analysis by subtype, sex, age, and diagnosis as shown in Table 3. Because adenocarcinoma is the most common pancreatic cancer subtype, it was used as the referent for subtype analysis. The prognoses of NETs, endocrinomas, lymphoma, and sarcoma were better than those of adenocarcinoma in univariate analysis. This remained significant in the multivariate analysis with an HR of 0.32 (95% CI, 0.29‐0.37; *P *< 1×10^−30^) for NETs, 0.28 (95% CI, 0.21‐0.36; *P *= 2×10^−20^) for endocrinomas, 0.47 (95% CI, 0.35‐0.63; *P *= 4×10^−7^) for lymphoma and 0.61 (95% CI, 0.40‐0.94; *P *= 0.025) for sarcoma after adjustment for sex, age, and diagnosis period. The survival rates of carcinoma, squamous cell carcinoma, and small cell carcinoma were worse than that of adenocarcinoma with a HRs of 1.34 (95% CI, 1.29‐1.38; *P *< 1×10^−30^), 1.80 (95% CI, 1.36‐2.40; *P *= 0.00005), and 1.42 (95% CI, 1.0‐2.0; *P *= 0.0496), respectively, in multivariate analysis. The survival of women was significantly better than that of men with an HR of 0.91 (95% CI, 0.88‐0.93; *P *= 2×10^−10^) in multivariate analysis. The prognosis of pancreatic cancer patients was poorer with increasing age, with those aged 80 years and older having the worst prognosis. We used T1 as the referent to evaluate whether the more recent diagnosis of pancreatic cancer (those diagnosed in T2) might have a better prognosis. The survival of pancreatic cancer in T2 was not better than that in T1 with an HR of 1 (95% CI, 0.97‐1.03; *P *= 0.828) for T2 according to multivariate analysis. When stratified by sex (Table S3), better survival of subtypes in both men and women was observed for NETs, endocrinomas, and lymphoma than that for survival of adenocarcinoma. The prognosis became poorer with increasing age for both men and women. The survival rate during T2 was better than that during T1 for women (HR = 0.95; 95% CI, 0.90‐0.99; *P *= 0.029) but not for men (HR = 1.04; 95% CI, 1.00‐1.08; *P *= 0.054).

**Table 3:** Cox proportional hazards survival analysis for pancreatic cancer by subtype, sex, age, and diagnosis period from 2002 to 2013

	Univariate			Multivariate		
	HR	95%CI	*P* value	HR	95%CI	*P* value
Subtypes Referent: adenocarcinoma						
Carcinoma	1.51	1.46‐1.56	<1 × 10^−30^	1.34	1.29‐1.38	<1 × 10^−30^
NETs	0.28	0.25‐0.32	<1 × 10^−30^	0.32	0.29‐0.37	<1 × 10^−30^
Endocrinomas	0.24	0.18‐0.31	1 × 10^−25^	0.28	0.21‐0.36	2 × 10^−20^
Lymphoma	0.41	0.31‐0.56	5 × 10^−9^	0.47	0.35‐0.63	4 × 10^−7^
Squamous cell carcinoma	1.71	1.29‐2.27	0.0002	1.8	1.36‐2.40	0.00005
Small cell carcinoma	1.50	1.06‐2.12	0.022	1.42	1.00‐2.00	0.05
Sarcoma	0.65	0.42‐1.00	0.05	0.61	0.40‐0.94	0.025
Sex Referent: Men						
Women	0.9	0.87‐0.93	6 × 10^−12^	0.91	0.88‐0.93	2 × 10^−10^
Age, years Referent: <30						
30 ≤ age < 40	2.72	2.01‐3.67	8 × 10^−11^	2.43	1.8‐3.29	7 × 10^−9^
40 ≤ age < 50	3.99	3.00‐5.29	1 × 10^−21^	3.39	2.55‐4.5	3 × 10^−17^
50 ≤ age < 60	4.41	3.33‐5.83	3 × 10^−25^	3.61	2.73‐4.78	3 × 10^−19^
60 ≤ age < 70	5.30	4.01‐7.00	<1 × 10^−30^	4.25	3.21‐5.62	3 × 10^−24^
70 ≤ age < 80	6.74	5.10‐8.91	<1 × 10^−30^	5.17	3.91‐6.83	<1 × 10^−30^
80 ≤ age	9.91	7.49‐13.11	<1 × 10^−30^	7.08	5.35‐9.37	<1 × 10^−30^
Diagnosed year Referent: 2002‐2007 (T1)						
2008‐2013 (T2)	0.94	0.91‐0.97	0.0001	1	0.97‐1.03	0.828

Because the diagnosis and treatment were updated over time, we evaluated the survival rate of pancreatic cancer patients by subtype during two time periods, 2002‐2007 (T1) and 2008‐2013 (T2) (Table S4). The 1‐year survival for all pancreatic cancers was 24.12% in T1 and 26.49% in T2. However, the 3‐year and 5‐year survival of all pancreatic cancers were all <10% during the two time periods. For adenocarcinoma, the most common subtype of pancreatic cancer, the 1‐year survival rates were 27.0% in T1 and 30.2% in T2. For carcinoma, the second most common type of pancreatic cancer, the 1‐year survival rates were 17.92% in T1 and 12.68% in T2. The 1‐year, 3‐year, and 5‐year survival rates of NETs were 61.9%, 38.1% and 23.8%, respectively, in T1 and 73.6%, 59.9%, and 50.6%, respectively, in T2. The 5‐year survival rate was 31.11% in T1 and increased to 50.95% in T2 in women. The 5‐year survival rate was only 15.38% in T1 in men, whereas it increased to 50.18% in T2, which was similar to that in women. The 1‐year, 3‐year, and 5‐year survival rates of lymphoma were 39.3%, 32.1%, and 28.6% in T1 and increased to 57.8%, 51.1%, and 51.1% in T2, respectively. We performed Cox proportional hazards survival analysis for each subtype of pancreatic cancer by sex, age, and diagnosis period as shown in Table 4. Improved survival from T1 to T2 was observed in adenocarcinoma (HR = 0.93; 95% CI, 0.89‐0.96; *P *= 0.0001; multivariate analysis), NETs (HR = 0.47; 95% CI, 0.35‐0.62; *P *= 1×10^−7^; multivariate analysis), and lymphoma (HR = 0.44; 95% CI, 0.22‐0.88; *P *= 0.02; multivariate analysis). Women displayed better survival than men for adenocarcinoma (HR = 0.88; 95% CI, 0.84‐0.91; *P *= 5×10^−11^; multivariate analysis) and carcinoma (HR = 0.92; 95% CI, 0.87‐0.96; *P *= 0.0006; multivariate analysis) but not for other subtypes.

**Table 4:** Cox proportional hazards survival analysis for subtypes of pancreatic cancers by sex, age, and diagnosis period from 2002 to 2013

	Adenocarcinoma						Carcinoma					
	Univariate			Multivariate			Univariate			Multivariate		
	HR	95% CI	*P* value	HR	95% CI	*P* value	HR	95% CI	*P* value	HR	95% CI	*P* value
Sex Referent: Men												
Women	0.86	0.83‐0.89	3 × 10^−14^	0.88	0.84‐0.91	5 × 10^−11^	0.98	0.94‐1.03	0.51	0.92	0.87‐0.96	0.0006
Age Referent: age<40												
40 ≤ age <50	1.85	1.6‐2.15	6 × 10^−16^	1.8	1.55‐2.08	2 × 10^−14^	1.62	1.26‐2.09	0.0002	1.6	1.25‐2.07	0.0002
50 ≤ age <60	1.91	1.66‐2.2	2 × 10^−19^	1.86	1.61‐2.14	7 × 10^−18^	1.78	1.41‐2.25	2 × 10^−6^	1.74	1.38‐2.2	4 × 10^−6^
60 ≤ age <70	2.15	1.87‐2.47	7 × 10^−27^	2.09	1.82‐2.4	5 × 10^−25^	2.3	1.82‐2.89	1 × 10^−12^	2.28	1.81‐2.87	2 × 10^−12^
70 ≤ age <80	2.59	2.25‐2.98	<1 × 10^−30^	2.52	2.19‐2.9	<1 × 10^−30^	2.71	2.16‐3.39	8 × 10^−18^	2.69	2.15‐3.38	1 × 10^−17^
80 ≤ age	3.73	3.23‐4.31	<1 × 10^−30^	3.64	3.15‐4.21	<1 × 10^−30^	3.44	2.74‐4.32	1 × 10^−26^	3.39	2.7‐4.25	7 × 10^−26^
Diagnosed year Referent: 2002‐2007 (T1)												
2008‐2013 (T2)	0.94	0.90‐0.97	0.001	0.93	0.89‐0.96	0.0001	1.18	1.12‐1.24	1 × 10^−10^	1.13	1.07‐1.18	5 × 10^−6^

	NETs						Endocrinomas					
	Univariate			Multivariate			Univariate			Multivariate		
	HR	95% CI	*P* value	HR	95% CI	*P* value	HR	95% CI	*P* value	HR	95% CI	*P* value
Sex Referent: Men												
Women	0.85	0.67‐1.09	0.197	0.87	0.68‐1.11	0.252	0.89	0.52‐1.53	0.669	0.94	0.53‐1.67	0.843
Age Referent: age<40												
40 ≤ age<50	1.38	0.84‐2.29	0.207	1.42	0.86‐2.36	0.173	0.9	0.34‐2.4	0.831	0.82	0.3‐2.28	0.705
50 ≤ age<60	1.59	1.00‐2.54	0.052	1.67	1.04‐2.67	0.033	0.87	0.33‐2.25	0.767	0.87	0.33‐2.3	0.78
60 ≤ age<70	2.03	1.28‐3.23	0.003	2.21	1.39‐3.52	0.0008	1.77	0.74‐4.22	0.197	1.82	0.75‐4.40	0.185
70 ≤ age<80	2.84	1.75‐4.59	0.00002	2.76	1.7‐4.49	0.00004	1.51	0.56‐4.06	0.41	1.46	0.52‐4.10	0.468
80 ≤ age	5.11	2.88‐9.05	2 × 10^−8^	6.13	3.43‐10.9	9 × 10^−10^	17.13	5.09‐57.7	4 × 10^−6^	15.1	4.33‐52.7	0.00002
Diagnosed year Referent: 2002‐2007 (T1)												
2008‐2013 (T2)	0.52	0.39‐0.68	3 × 10^−6^	0.47	0.35‐0.62	1 × 10^−7^	0.73	0.41‐1.28	0.268	0.75	0.41‐1.38	0.361

	Lymphoma						Sarcoma					
	Univariate			Multivariate			Univariate			Multivariate		
	HR	95% CI	*P* value	HR	95% CI	*P* value	HR	95% CI	*P* value	HR	95% CI	*P* value
Sex Referent: Men												
Women	1.27	0.70‐2.3	0.438	1.03	0.54‐1.99	0.923	0.9	0.38‐2.16	0.82	0.87	0.32‐2.38	0.788
Age Referent: age<40							Referent: age <50					
40 ≤ age<50	2.37	0.25‐22.8	0.455	2.16	0.22‐20.8	0.506						
50 ≤ age<60	3.47	0.43‐27.8	0.241	3.14	0.39‐25.2	0.281	2.37	0.29‐19.4	0.421	2.32	0.28‐19.6	0.439
60 ≤ age<70	10.7	1.32‐87.4	0.027	13.7	1.6‐114.1	0.016	3.66	0.4‐33.3	0.25	2.88	0.25‐32.8	0.395
70 ≤ age<80	10.9	1.43‐83.4	0.021	9.14	1.18‐70.8	0.034	5.63	0.64‐49.9	0.121	5.41	0.56‐52.4	0.146
80 ≤ age	25.7	3.3‐203.6	0.002	28.6	3.6‐229.6	0.002	1.56	0.16‐15.6	0.705	1.45	0.14‐15.2	0.758
Diagnosed year Referent: 2002‐2007 (T1)												
2008‐2013 (T2)	0.58	0.32‐1.06	0.078	0.44	0.22‐0.88	0.02	0.67	0.28‐1.60	0.37	0.83	0.29‐2.36	0.72

	Squamous cell carcinoma						Small cell carcinoma					
	Univariate			Multivariate			Univariate			Multivariate		
	HR	95% CI	*P* value	HR	95% CI	*P* value	HR	95% CI	*P* value	HR	95% CI	*P* value
Sex Referent: Men												
Women	0.73	0.40‐1.33	0.305	0.71	0.36‐1.40	0.319	0.91	0.42‐1.99	0.813	0.94	0.39‐2.25	0.888
Age Referent: age<40												
40 ≤ age<50	0.51	0.10‐2.74	0.433	0.35	0.06‐2.11	0.254	0.9	0.06‐14.6	0.943	0.9	0.06‐14.6	0.943
50 ≤ age<60	0.46	0.09‐2.26	0.341	0.29	0.05‐1.67	0.164	0.61	0.07‐5.26	0.649	0.63	0.07‐5.6	0.68
60 ≤ age<70	0.47	0.10‐2.15	0.33	0.33	0.06‐1.68	0.181	0.37	0.05‐3.02	0.355	0.39	0.05‐3.18	0.376
70 ≤ age<80	1.27	0.28‐5.64	0.757	0.85	0.17‐4.3	0.841	0.44	0.05‐3.78	0.451	0.46	0.05‐4.04	0.48
80 ≤ age	1.71	0.31‐9.41	0.536	1.04	0.16‐6.78	0.965	0.55	0.06‐5.18	0.604	0.57	0.06‐5.39	0.624
Diagnosed year Referent: 2002‐2007 (T1)												
2008‐2013 (T2)	1.27	0.72‐2.26	0.413	1.32	0.71‐2.45	0.374	1.19	0.53‐2.66	0.68	1.08	0.44‐2.65	0.869

Table 5 presents the survival analysis for pancreatic cancer by subtype, sex, and age during the two time periods (T1 and T2). The prognoses of NETs, endocrinomas, and lymphoma were better than that of adenocarcinoma in T1 (NETs: HR = 0.62, 95% CI = 0.49‐0.77, *P *= 3×10^−5^; endocrinomas: HR = 0.33, 95% CI = 0.23‐0.47, *P *= 3×10^−10^; lymphoma: HR = 0.65, 95% CI = 0.42‐1.00, *P *= 0.048) and T2 (NETs: HR = 0.27, 95% CI = 0.24‐0.32, *P *< 1×10^−30^; endocrinomas: HR = 0.22, 95% CI = 0.14‐0.34, *P *= 9×10^−12^; lymphoma: HR = 0.37, 95% CI = 0.25‐0.56, *P *= 2×10^−6^) by multivariate analysis. The prognosis of sarcoma was not significantly different from that of adenocarcinoma in T1 (HR = 0.73; 95% CI, 0.40‐1.31; *P *= 0.288) but was better than that of adenocarcinoma in T2 (HR = 0.51; 95% CI, 0.27‐0.95; *P *= 0.034) by multivariate analysis. Compared with men, the risk of death in women was consistently lower. The HR was 0.95 (95% CI, 0.91‐0.99; *P *= 0.027) in T1 and 0.87 (95% CI, 0.84‐0.91; *P *= 2×10^−11^) in T2 for women by multivariate analysis. Additionally, there was a similar increasing trend in the risk of death with age for the two time periods.

**Table 5:** Cox proportional hazards survival analysis for pancreatic cancer patients by subtype, sex, and age in two time periods

	2002‐2007						2008‐2013					
	Univariate			Multivariate			Univariate			Multivariate		
	HR	95% C.I.	*P* value	HR	95% C.I.	*P* value	HR	95% C.I.	*P* value	HR	95% C.I.	*P* value
Subtypes Referent: adenocarcinoma												
Carcinoma	1.3	1.24‐1.37	8 × 10^−28^	1.18	1.12‐1.23	1 × 10^−10^	1.73	1.65‐1.80	<1 × 10^−30^	1.5	1.43‐1.57	<1 × 10^−30^
NETs	0.51	0.41‐0.64	8 × 10^−9^	0.62	0.49‐0.77	3 × 10^−5^	0.24	0.21‐0.28	<1 × 10^−30^	0.27	0.24‐0.32	<1 × 10^−30^
Endocrinomas	0.29	0.20‐0.40	8 × 10^−13^	0.33	0.23‐0.47	3 × 10^−10^	0.18	0.12‐0.28	2 × 10^−14^	0.22	0.14‐0.34	9 × 10^−12^
Lymphoma	0.57	0.37‐0.88	0.01	0.65	0.42‐1.00	0.048	0.33	0.22‐0.50	1 × 10^−7^	0.37	0.25‐0.56	2 × 10^−6^
Squamous cell carcinoma	1.44	0.94‐2.18	0.09	1.55	1.02‐2.36	0.04	2.01	1.37‐2.96	0.0004	2.07	1.41‐3.05	0.0002
Small cell carcinoma	1.3	0.65‐2.60	0.46	1.24	0.62‐2.49	0.54	1.6	1.07‐2.39	0.021	1.49	1.00‐2.23	0.05
Sarcoma	0.87	0.48‐1.56	0.63	0.73	0.40‐1.31	0.288	0.51	0.27‐0.94	0.032	0.51	0.27‐0.95	0.034
Sex Referent: Male												
Female	0.93	0.89‐0.97	0.002	0.95	0.91‐0.99	0.027	0.88	0.84‐0.92	3 × 10^−10^	0.87	0.84‐0.91	2 × 10^−11^
Age, years Referent: age <30												
30 ≤ age<40	2.83	1.80‐4.43	6 × 10^−6^	2.76	1.76‐4.33	0.00001	2.6	1.73‐3.89	4 × 10^−6^	2.14	1.43‐3.21	0.0002
40 ≤ age<50	3.71	2.42‐5.68	2 × 10^−9^	3.54	2.31‐5.42	6 × 10^−9^	4.17	2.85‐6.08	1 × 10^−13^	3.21	2.19‐4.68	2 × 10^−9^
50 ≤ age<60	4.06	2.66‐6.19	8 × 10^−11^	3.79	2.48‐5.78	6 × 10^−10^	4.68	3.22‐6.79	6 × 10^−16^	3.38	2.33‐4.92	2 × 10^−10^
60 ≤ age<70	5.04	3.31‐7.68	5 × 10^−14^	4.65	3.05‐7.09	9 × 10^−13^	5.48	3.77‐7.95	4 × 10^−19^	3.87	2.66‐5.62	1 × 10^−12^
70 ≤ age<80	6.18	4.06‐9.41	2 × 10^−17^	5.61	3.68‐8.54	1 × 10^−15^	7.13	4.91‐10.34	5 × 10^−25^	4.73	3.25‐6.86	4 × 10^−16^
80 ≤ age	8.87	5.81‐13.53	4 × 10^−24^	7.69	5.04‐11.74	3 × 10^−21^	10.72	7.38‐15.57	<1 × 10^−30^	6.43	4.42‐9.36	2 × 10^−22^

Taken together, the best survival of pancreatic cancer was observed in those with endocrinomas, NETs, and lymphoma, and the worst survival was observed in those with small cell carcinoma, squamous cell carcinoma, carcinoma, and adenocarcinoma. The overall survival was significantly prolonged in the recent decade for patients with adenocarcinoma, NETs, and lymphoma.

## 4 DISCUSSION

We observed several changes in the incidence and survival rates of pancreatic cancer in this nation‐wide population‐based study in Taiwan. The incidence of all pancreatic cancers increased from 4.62 per 100 000 in 2002 to 6.04 per 100 000 in 2013. The incidence of NETs showed the most significant increase. The survival rates of adenocarcinoma, NETs, and lymphoma significantly improved from 2002 to 2013.

The incidence of pancreatic cancer varies across regions and populations. The incidence of pancreatic cancer in Asia was lower than that in Northern America and Western Europe but more than that in Middle Africa.^1^ According to the estimation by the IARC for Asia in 2012, the age‐standardized incidence rate was 3.2 per 100 000.^10^ In Taiwan, the age‐standardized incidence rate was 5.76 per 100 000 in 2012. The increased trend was also noted with an APC of 2.6. The increased trend of pancreatic cancer was also observed in China with an APC of 5.29 from 2003 to 2009 and in Iran with the age‐standardized incidence rate increasing from 0.75 per 100 000 in 2001 to 2.68 per 100 000 in 2011.^4^, ^11^ The age‐standardized incidence rate of pancreatic cancer in China in 2009 was 4.63 per 100 000. By contrast, the age‐standardized incidence rate of pancreatic cancer in Taiwan was 5.61 per 100 000 in 2009 and 5.76 per 100 000 in 2012, rates that were significantly higher than that of pancreatic cancer in China and that estimated by the IARC for Asia. From our data, the increased incidence rate of pancreatic cancer was only observed in adenocarcinoma and NETs for both men and women. The overall increased incidence of pancreatic cancer was mainly due to the increase in adenocarcinoma because the increased trends were similar between all pancreatic cancers and adenocarcinoma as shown in Figure 1A,B. Although the incidence of NETs was also significantly increased, the increase did not affect the overall increase in pancreatic cancer significantly due to the relatively lower percentage. However, the relatively stable incidence of other subtypes, including endocrinomas, lymphoma, squamous cell carcinoma, small cell carcinoma, and sarcoma, suggested the presence of different etiologies for adenocarcinoma, NETs, and other subtypes of pancreatic cancer. Many of the NETs were probably misdiagnosed as pancreatic carcinoma or adenocarcinoma previously. The increased incidence of NETs may be explained partially by the more accurate diagnosis due to the increased awareness of NETs by physicians after the establishment of pancreatic NET classification in 2000 by WHO and the update in 2010.^12^ The risk factors for pancreatic adenocarcinoma include a history of pancreatitis, cigarette smoking, diabetes, dietary factors, infection (infection with hepatitis B virus, hepatitis C virus or *Helicobacter pylori*), or genetic factors.^13^, ^14^ However, the risk factors for pancreatic NETs is unclear. A family history of cancer, diabetes, alcohol, smoking, and chronic pancreatitis has been reported to be risk factors for pancreatic NETs in several case‐control studies. Among these risk factors, diabetes and a family history of cancer were more consistently observed by the studies.^15^, ^16^, ^17^, ^18^, ^19^, ^20^ The increase in adenocarcinoma and NETs has not reached a plateau, suggesting the importance of identifying and reducing the risk factors to prevent a further increase in the incidence of pancreatic cancer, particularly adenocarcinoma and NETs.

In the survival analysis, we observed that the survival rates of adenocarcinoma, NETs, and lymphoma improved in the recent decade. Adenocarcinoma and carcinoma accounted for 95.6% of all pancreatic cancers. The survival of these patients was poor. Only 15%‐20% of pancreatic cancer patients have resectable disease, and the 5‐year survival rate of those patients is approximately 20%.^21^, ^22^ According to the annual report of the TCR, only 20.8% of pancreatic cancer patients received surgery from 2003 to 2009.^23^ Most of the cases were diagnosed at advanced stages. Chemotherapy is the main treatment strategy for advanced pancreatic adenocarcinoma and carcinoma. Gemcitabine is commonly used as first‐line treatment for advanced pancreatic adenocarcinoma or carcinoma since 1997.^24^ The combination of gemcitabine with other agents, such as erlotinib, S‐1, or abraxane^25^, ^26^, ^27^, ^28^, ^29^, or the use of other chemotherapeutic agents, such as oxaliplatin, irinotecan, fluoropyrimidine, and S‐1, has been shown to prolong the progression‐free survival and overall survival of pancreatic cancer patients in recent years.^30^, ^31^ However, the median overall survival is still <1 year. Furthermore, old age at diagnosis is another factor to limit the use of chemotherapy. In our study, around one‐half of the patients were diagnosed at more than 70 years of age, limiting the use of chemotherapy. Thus, only 36.7% of pancreatic cancer patients received chemotherapy from 2003 to 2009 in Taiwan according to the TCR annual reports.^23^^.^

Lymphoma accounted for only 0.4% of all pancreatic cancers in Taiwan. However, the survival of lymphoma has significantly improved in the recent decade. Approximately 7% of lymphomas are Hodgkin's lymphoma, and 93% are non‐Hodgkin's lymphoma (NHL).^23^ Approximately 80% of NHLs are B‐cell lymphomas, and diffuse large B‐cell lymphoma (DLBCL) is the most common type of lymphoma in the western countries or Asia.^32^, ^33^ Treatment for lymphoma did not improve significantly until the introduction of rituximab, an anti‐CD20 monoclonal antibody. The 5‐year overall survival rate of DLBCL increased to 58% with the addition of rituximab to CHOP versus 45% for elderly patients treated with CHOP only.^34^ Rituximab has been shown to prolong PFS in relapsed low‐grade NHL patients in 1997^35^ and was approved as the first‐line treatment in DLBCL in 2006.^36^ In Taiwan, rituximab use has been reimbursed for refractory low‐grade lymphoma since April, 2002 and for DLBCL since January, 2004 by the Bureau of National Health Insurance (BNHI). Although the exact distribution of lymphoma types in our patient population is unknown, we believe that the addition of rituximab is the major contribution for the increased survival of pancreatic lymphoma patients in T2.

The 1‐year survival rate of adenocarcinoma was increased in both men and women from T1 to T2 (Table S2), suggesting that new chemotherapy regimens are beneficial for both men and women. However, why the survival rate for adenocarcinoma in women is better than that in men remains unknown. For the survival analysis of other pancreatic cancer subtypes, we noticed that the 5‐year survival rate for NETs was significantly better in women than in men in T1. However, the survival rates of pancreatic NETs in women and men improved and were similar in T2. This might be due to the introduction of new therapies for pancreatic NETs. Surgery can usually cure pancreatic NETs in the early stages. However, most cases of pancreatic NETs are diagnosed at an advanced stage due to the lack of clinical symptoms and signs. Treatment for advanced pancreatic NETs comprised chemotherapy with streptozocin, doxorubicin, or fluoropyrimidine‐based regimens prior to the introduction of targeted therapies. Targeted agents, including everolimus and sunitinib, were shown to improve the progression‐free survival for advanced pancreatic NETs in phase III trials.^37^, ^38^ These two agents were approved by the FDA in May, 2011. The reimbursement for sunitinib and everolimus in Taiwan by the BNHI began in May 2012 and January 2013, respectively. Therefore, it is possible that the improvement in the survival rate of pancreatic NETs in T2 was due to the introduction of new therapies for pancreatic NETs. In summary, the incidence of pancreatic cancer in Taiwan, particularly that of adenocarcinoma and NETs in both men and women, has been increasing rapidly. The survival rates of adenocarcinoma, lymphoma, and NET patients have improved probably due to the introduction of new chemotherapy regimens and targeted therapies. Despite the improvement in survival, it is important to identify and reduce the risk factors for pancreatic cancer to prevent the development of pancreatic cancer. It is also important to develop biomarkers for the early detection of pancreatic cancer and establish novel therapies for the treatment of pancreatic cancer to improve the survival of pancreatic cancer.

## CONFLICTS OF INTEREST

The authors declare no competing financial interests.

## References

[1] Ilic M , Ilic I . Epidemiology of pancreatic cancer. World J Gastroenterol. 2016;22(44):9694‐9705. 27956793 10.3748/wjg.v22.i44.9694 PMC5124974

[2] Health Promotion Administration, Ministry of Health and Welfare, the Executive Yuan, Taiwan. Cancer Registry Annual Report, Taiwan. Available on http://www.hpa.gov.tw/ Accessed on December 9, 2016.

[3] Jung KW , Park S , Kong HJ , et al. Cancer statistics in Korea: incidence, mortality and survival in 2006‐2007. J Korean Med Sci. 2010;25(8):1113‐1121. 20676319 10.3346/jkms.2010.25.8.1113 PMC2908777

[4] Chen WQ , Liang D , Zhang SW , Zheng RS , He YT . Pancreatic cancer incidence and mortality patterns in China, 2009. Asian Pac J Cancer Prev. 2013;14(12):7321‐7324. 24460295 10.7314/apjcp.2013.14.12.7321

[5] Luo J , Xiao L , Wu C , Zheng Y , Zhao N . The incidence and survival rate of population‐based pancreatic cancer patients: Shanghai Cancer Registry 2004‐2009. PLoS ONE. 2013;8(10):e76052. 24130758 10.1371/journal.pone.0076052 PMC3794034

[6] Egawa S , Toma H , Ohigashi H , et al. Japan Pancreatic Cancer Registry; 30th year anniversary Japan Pancreas Society. Pancreas. 2012;41(7):985‐992. 22750974 10.1097/MPA.0b013e318258055c

[7] Hong Kong Cancer Registry. http://www3.ha.org.hk/cancereg/allages.asp. Accessed July 27th, 2018.

[8] International Agency for Research on Cancer. GLOBOCAN 2012: Estimated Cancer Incidence, Mortality and Prevalence Worldwide in 2012. http://globocan.iarc.fr/Default.aspx. Accessed July 27th, 2018.

[9] Tsai HJ , Wu CC , Tsai CR , Lin SF , Chen LT , Chang JS . The epidemiology of neuroendocrine tumors in Taiwan: a nation‐wide registry‐based study. PLoS ONE. 2013;8(4):e62487. 23614051 10.1371/journal.pone.0062487 PMC3632554

[10] Pourhoseingholi MA , Vahedi M , Baghestani AR . Burden of gastrointestinal cancer in Asia; an overview. Gastroenterol Hepatol Bed Bench. 2015;8(1):19‐27. 25584172 PMC4285928

[11] Zahir ST , Arjmand A , Kargar S , Neishaboury M . Incidence and trends of malignant and benign pancreatic lesions in Yazd, Iran between 2001 and 2011. Asian Pac J Cancer Prev. 2013;14(4):2631‐2635. 23725187 10.7314/apjcp.2013.14.4.2631

[12] Oberg KE . The management of neuroendocrine tumours: current and future medical therapy options. Clin Oncol. 2012;24(4):282‐293. 10.1016/j.clon.2011.08.006 21907552

[13] Maisonneuve P , Lowenfels AB . Risk factors for pancreatic cancer: a summary review of meta‐analytical studies. Int J Epidemiol. 2015;44(1):186‐198. 25502106 10.1093/ije/dyu240

[14] Wörmann SM , Algül H . Risk factors and therapeutic targets in pancreatic cancer. Front Oncol. 2013;3:282. 24303367 10.3389/fonc.2013.00282 PMC3831165

[15] Hassan MM , Phan A , Li D , Leary C , Yao JC . Risk factors associated with neuroendocrine tumors: a U.S.‐based case‐control study. Int J Cancer. 2008;123(4):867‐873. 18491401 10.1002/ijc.23529

[16] Halfdanarson TR , Bamlet WR , McWilliams RR , et al. Risk factors for pancreatic neuroendocrine tumors: a clinic‐based case‐control study. Pancreas. 2014;43(8):1219‐1222. 25291526 10.1097/MPA.0000000000000234 PMC4267883

[17] Capurso G , Falconi M , Panzuto F , et al. Risk factors for sporadic pancreatic endocrine tumors: a case‐control study of prospectively evaluated patients. Am J Gastroenterol. 2009;104(12):3034‐3041. 19690522 10.1038/ajg.2009.466

[18] Ben Q , Zhong J , Fei J , et al. Risk factors for sporadic pancreatic neuroendocrine tumors: a case‐control study. Sci Rep. 2016;6:36073. 27782199 10.1038/srep36073 PMC5080551

[19] Haugvik SP , Hedenström P , Korsæth E , et al. Diabetes, smoking, alcohol use, and family history of cancer as risk factors for pancreatic neuroendocrine tumors: a systematic review and meta‐analysis. Neuroendocrinology. 2015;101(2):133‐142. 25613442 10.1159/000375164

[20] Valente R , Hayes AJ , Haugvik SP , et al. Risk and protective factors for the occurrence of sporadic pancreatic endocrine neoplasms. Endocr Relat Cancer. 2017;24(8):405‐414. 28566532 10.1530/ERC-17-0040

[21] Yeo CJ , Cameron JL , Lillemoe KD , et al. Pancreaticoduodenectomy for cancer of the head of the pancreas. 201 patients. Ann Surg. 1995;221(6):721‐731. 7794076 10.1097/00000658-199506000-00011 PMC1234702

[22] Li D , Xie K , Wolff R , Abbruzzese JL . Pancreatic cancer. Lancet. 2004;363(9414):1049‐1057. 15051286 10.1016/S0140-6736(04)15841-8

[23] Taiwan Cancer Registry, annual report. Available from: http://tcr.cph.ntu.edu.tw/main.php?Page=A5. Accessed on November 2, 2017

[24] Burris HA III , Moore MJ , Andersen J , et al. Improvements in survival and clinical benefit with gemcitabine as first‐line therapy for patients with advanced pancreas cancer: a randomized trial. J Clin Oncol. 1997;15(6):2403‐2413. 9196156 10.1200/JCO.1997.15.6.2403

[25] Moore MJ , Goldstein D , Hamm J , et al. Erlotinib plus gemcitabine compared with gemcitabine alone in patients with advanced pancreatic cancer: a phase III trial of the National Cancer Institute of Canada Clinical Trials Group. J Clin Oncol. 2007;25(15):1960‐1966. 17452677 10.1200/JCO.2006.07.9525

[26] Oh DY , Cha Y , Choi IS , et al. A multicenter phase II study of gemcitabine and S‐1 combination chemotherapy in patients with unresectable pancreatic cancer. Cancer Chemother Pharmacol. 2010;65(3):527‐536. 19578850 10.1007/s00280-009-1059-9

[27] Ueno H , Okusaka T , Furuse J , et al. Multicenter phase II study of gemcitabine and S‐1 combination therapy (GS therapy) in patients with metastatic pancreatic cancer. Jpn J Clin Oncol. 2011;41(8):953‐958. 21715364 10.1093/jjco/hyr090

[28] Ueno H , Ioka T , Ikeda M , et al. Randomized phase III study of gemcitabine plus S‐1, S‐1 alone, or gemcitabine alone in patients with locally advanced and metastatic pancreatic cancer in Japan and Taiwan: GEST study. J Clin Oncol. 2013;31(13):1640‐1648. 23547081 10.1200/JCO.2012.43.3680

[29] Von Hoff DD , Ervin T , Arena FP , et al. Increased survival in pancreatic cancer with nab‐paclitaxel plus gemcitabine. N Engl J Med. 2013;369(18):1691‐1703. 24131140 10.1056/NEJMoa1304369 PMC4631139

[30] Conroy T , Desseigne F , Ychou M , et al. FOLFIRINOX versus gemcitabine for metastatic pancreatic cancer. N Engl J Med. 2011;364(19):1817‐1825. 21561347 10.1056/NEJMoa1011923

[31] Takahara N , Isayama H , Nakai Y , et al. A retrospective study of S‐1 and oxaliplatin combination chemotherapy in patients with refractory pancreatic cancer. Cancer Chemother Pharmacol. 2013;72(5):985‐990. 23995699 10.1007/s00280-013-2278-7

[32] Morton LM , Wang SS , Devesa SS , Hartge P , Weisenburger DD , Linet MS . Lymphoma incidence patterns by WHO subtype in the United States. Blood. 2006;107(1):265‐276. 16150940 10.1182/blood-2005-06-2508 PMC1895348

[33] Carreon JD , Morton LM , Devesa SS , et al. Incidence of lymphoid neoplasms by subtype among six Asia ethnic groups in the United States, 1996‐2004. Cancer Causes Control. 2008;19(10):1171‐1181. 18543071 10.1007/s10552-008-9184-z PMC2581633

[34] Feugier P , Van Hoof A , Sebban C , et al. Long term results of the R‐CHOP study in the treatment of elderly patients with diffuse large B cell lymphoma: a study by the groupe d'Etude des Lymphomes del'Adulte. J Clin Oncol. 2005;23(18):4117‐4126. 15867204 10.1200/JCO.2005.09.131

[35] Maloney DG , Grillo‐López AJ , White CA , et al. IDEC‐C2B8 (Rituximab) anti‐CD20 monoclonal antibody therapy in patients with relapsed low‐grade non‐Hodgkin's lymphoma. Blood. 1997;90(6):2188‐2195. 9310469

[36] National Cancer Institute, FDA approval for Rituximab. Available from: https://www.cancer.gov/about-cancer/treatment/drugs/fda-rituximab. Accessed on November 2, 2017.

[37] Yao JC , Shah MH , Ito T , et al. Everolimus for advanced pancreatic neuroendocrine tumors. N Engl J Med. 2011;364(6):514‐523. 21306238 10.1056/NEJMoa1009290 PMC4208619

[38] Raymond E , Dahan L , Raoul JL , et al. Sunitinib malate for the treatment of pancreatic neuroendocrine tumors. N Engl J Med. 2011;364(6):501‐513. 21306237 10.1056/NEJMoa1003825

