# Supplementary material for: The incidence and survival of pancreatic cancer by histology, including rare subtypes: a nation‐wide cancer registry‐based study from Taiwan
Source: Cancer Med. 2018 Sep 27;7(11):5775–88. doi: 10.1002/cam4.1795 (PMC6246938; doi:10.1002/cam4.1795)
Supplement: Supplementary file 1 [file CAM4-7-5775-s001.docx]

**Supplementary Table 1.** The incidence and mortality of pancreatic cancer in Asia in 2012

|  | Incidence | Mortality |
| --- | --- | --- |
|  | ASR*  (cases per 100,000) | ASR*  (cases per 100,000) |
| Japan | 8.5 | 7.7 |
| Korea | 6.7 | 6.2 |
| Taiwan | 5.8 | 4.8 |
| China | 3.6 | 3.5 |
| Hong Kong | 4.1 | 3.7 |
| Singapore | 5.1 | 5.6 |
| Indonesia | 2.7 | 2.6 |
| Malaysia | 2.4 | 3.2 |
| Thailand | 2.1 | 1.8 |
| India | 1.2 | 1.1 |
| Vietnam | 1 | 1 |
| Bangladesh | 0.7 | 0.7 |

*ASR, age-standardized rate
