# Supplementary material for: The incidence and survival of pancreatic cancer by histology, including rare subtypes: a nation‐wide cancer registry‐based study from Taiwan
Source: Cancer Med. 2018 Sep 27;7(11):5775–88. doi: 10.1002/cam4.1795 (PMC6246938; doi:10.1002/cam4.1795)
Supplement: Supplementary file 2 [file CAM4-7-5775-s002.doc]

**Supplementary Table 2**. The annual age-standardized incidence rate (cases per 100,000) of pancreatic cancer in Taiwan from 2002 to 2013 by subtype, sex, and the year of diagnosis

|  | **Year of diagnosis** | | | | | | | | | | | | **APC** | **p-value** |
| --- | --- | --- | --- | --- | --- | --- | --- | --- | --- | --- | --- | --- | --- | --- |
|  | **2002** | **2003** | **2004** | **2005** | **2006** | **2007** | **2008** | **2009** | **2010** | **2011** | **2012** | **2013** |  |  |
| **Overall** | **4.62** | **4.75** | **4.61** | **4.61** | **5.12** | **5.07** | **5.24** | **5.61** | **5.38** | **5.79** | **5.76** | **6.04** | **2.6** | **1x10^-6^** |
| Men | 5.29 | 5.53 | 5.27 | 5.12 | 6.19 | 6.09 | 6.17 | 6.75 | 6.31 | 6.80 | 6.79 | 6.99 | 2.85 | 0.00004 |
| Women | 3.96 | 3.94 | 3.95 | 4.10 | 4.07 | 4.09 | 4.34 | 4.53 | 4.50 | 4.84 | 4.81 | 5.16 | 2.46 | 1x10^-6^ |
| **Adenocarcinoma** | **2.61** | **2.69** | **2.71** | **2.58** | **2.96** | **3.01** | **3.21** | **3.46** | **3.39** | **3.64** | **3.65** | **3.87** | **3.94** | **2x10^-7^** |
| Men | 3.05 | 3.07 | 3.09 | 2.86 | 3.60 | 3.64 | 3.82 | 4.31 | 3.95 | 4.20 | 4.39 | 4.69 | 4.4 | 7x10^-6^ |
| Women | 2.16 | 2.30 | 2.32 | 2.30 | 2.31 | 2.41 | 2.63 | 2.65 | 2.86 | 3.12 | 2.96 | 3.11 | 3.54 | 7x10^-7^ |
| **Carcinoma** | **1.89** | **1.95** | **1.80** | **1.91** | **2.01** | **1.88** | **1.84** | **1.92** | **1.74** | **1.82** | **1.65** | **1.68** | **-1.14** | **0.01** |
| Men | 2.14 | 2.33 | 2.10 | 2.14 | 2.42 | 2.25 | 2.17 | 2.21 | 2.07 | 2.22 | 1.87 | 1.81 | -1.37 | 0.04 |
| Women | 1.64 | 1.55 | 1.50 | 1.68 | 1.62 | 1.52 | 1.52 | 1.65 | 1.42 | 1.45 | 1.45 | 1.55 | -0.77 | 0.09 |
| **NETs** | **0.02** | **0.02** | **0.05** | **0.05** | **0.08** | **0.10** | **0.10** | **0.11** | **0.16** | **0.25** | **0.39** | **0.43** | **31.52** | **3x10^-8^** |
| Men | 0.02 | 0.04 | 0.02 | 0.04 | 0.08 | 0.10 | 0.10 | 0.06 | 0.20 | 0.27 | 0.45 | 0.41 | 34.02 | 3x10^-8^ |
| Women | 0.02 | 0.01 | 0.09 | 0.06 | 0.09 | 0.10 | 0.10 | 0.15 | 0.13 | 0.23 | 0.33 | 0.45 | 32.48 | 0.00004 |
| **Endocrinomas** | **0.05** | **0.05** | **0.03** | **0.03** | **0.01** | **0.04** | **0.05** | **0.05** | **0.03** | **0.02** | **0.01** | **0.01** | **-10.03** | **0.06** |
| Men | 0.04 | 0.03 | 0.03 | 0.02 | 0.01 | 0.06 | 0.04 | 0.06 | 0.01 | 0.02 | 0.01 | 0.01 | -10.32 | 0.1 |
| Women | 0.07 | 0.06 | 0.02 | 0.04 | 0.02 | 0.02 | 0.07 | 0.04 | 0.05 | 0.03 | 0.02 | 0.01 | -11.1 | 0.06 |
| **Lymphoma** | **0.02** | **0.02** | **0.01** | **0.02** | **0.03** | **0.02** | **0.02** | **0.03** | **0.02** | **0.02** | **0.03** | **0.03** | **4.56** | **0.06** |
| Men | 0.00 | 0.02 | 0.01 | 0.03 | 0.04 | 0.02 | 0.01 | 0.05 | 0.04 | 0.02 | 0.03 | 0.03 | 6.67 | 0.21 |
| Women | 0.04 | 0.02 | 0.02 | 0.00 | 0.02 | 0.01 | 0.03 | 0.02 | 0.00 | 0.01 | 0.03 | 0.02 | -1.03 | 0.78 |
| **Squamous cell carcinoma** | **0.03** | **0.01** | **0.00** | **0.02** | **0.02** | **0.01** | **0.00** | **0.02** | **0.02** | **0.02** | **0.02** | **0.01** | **2.76** | **0.68** |
| Men | 0.02 | 0.01 | 0.01 | 0.03 | 0.02 | 0.00 | 0.01 | 0.03 | 0.02 | 0.03 | 0.03 | 0.01 | 2.54 | 0.65 |
| Women | 0.03 | 0.00 | 0.00 | 0.01 | 0.03 | 0.02 | 0.00 | 0.01 | 0.01 | 0.01 | 0.01 | 0.01 | -10.38 | 0.04 |
| **Small cell carcinoma** | **0.004** | **0.01** | **0.004** | **0.004** | **0.003** | **0.01** | **0.01** | **0.02** | **0.01** | **0.02** | **0.01** | **0.02** | **15.23** | **0.002** |
| Men | 0.00 | 0.01 | 0.00 | 0.00 | 0.01 | 0.01 | 0.03 | 0.03 | 0.01 | 0.03 | 0.01 | 0.03 | 9.67 | 0.18 |
| Women | 0.01 | 0.00 | 0.01 | 0.01 | 0.00 | 0.01 | 0.00 | 0.01 | 0.01 | 0.00 | 0.01 | 0.01 | -0.5 | 0.8 |
| **Sarcoma** | **0.01** | **0.02** | **0.00** | **0.01** | **0.01** | **0.00** | **0.00** | **0.01** | **0.02** | **0.00** | **0.01** | **0.01** | **-2.8** | **0.57** |
| Men | 0.02 | 0.02 | 0.00 | 0.01 | 0.01 | 0.00 | 0.00 | 0.01 | 0.02 | 0.01 | 0.01 | 0.01 | -9.44 | 0.03 |
| Women | 0.00 | 0.02 | 0.01 | 0.01 | 0.00 | 0.00 | 0.00 | 0.01 | 0.02 | 0.00 | 0.01 | 0.01 | -0.67 | 0.9 |
