# Supplementary material for: The incidence and survival of pancreatic cancer by histology, including rare subtypes: a nation‐wide cancer registry‐based study from Taiwan
Source: Cancer Med. 2018 Sep 27;7(11):5775–88. doi: 10.1002/cam4.1795 (PMC6246938; doi:10.1002/cam4.1795)
Supplement: Supplementary file 3 [file CAM4-7-5775-s003.docx]

**Supplementary Table 3.** Cox proportional hazards survival analysis for pancreatic cancers by subtypes, age and diagnosed period in men and women from 2002 to 2013.

|  | Men | | | | | | Women | | | | | |
| --- | --- | --- | --- | --- | --- | --- | --- | --- | --- | --- | --- | --- |
|  | Univariate | | | Multivariate | | | Univariate | | | Multivariate | | |
|  | HR | 95%CI | *P* value | HR | 95%CI | *P* value | HR | 95%CI | *P* value | HR | 95%CI | *P* value |
| **Subtypes**  Referent: adenocarcinoma | | | | | | | | | | | | |
| carcinoma | 1.42 | 1.36-1.48 | <1x10^-30^ | 1.32 | 1.26-1.38 | <1x10^-30^ | 1.65 | 1.57-1.73 | 4x10^-30^ | 1.35 | 1.29-1.43 | 1x10^-30^ |
| NETs | 0.29 | 0.24-0.34 | <1x10^-30^ | 0.32 | 0.27-0.37 | <1x10^-30^ | 0.28 | 0.23-0.33 | <1x10^-30^ | 0.33 | 0.28-0.40 | <1x10^-30^ |
| endocrinomas | 0.25 | 0.17-0.37 | 5x10^-12^ | 0.27 | 0.18-0.40 | 6x10^-11^ | 0.23 | 0.16-0.34 | 1x10^-14^ | 0.29 | 0.20-0.42 | 9x10^-11^ |
| lymphoma | 0.36 | 0.24-0.53 | 3x10^-7^ | 0.41 | 0.28-0.61 | 1x10^-5^ | 0.51 | 0.33-0.81 | 0.004 | 0.53 | 0.34-0.84 | 0.006 |
| squamous cell carcinoma | 1.8 | 1.27-2.55 | 0.0009 | 1.91 | 1.35-2.70 | 0.0003 | 1.53 | 0.93-2.49 | 0.091 | 1.63 | 1.00-2.66 | 0.052 |
| small cell carcinoma | 1.41 | 0.93-2.12 | 0.102 | 1.37 | 0.91-2.07 | 0.128 | 1.64 | 0.85-3.16 | 0.137 | 1.47 | 0.76-2.83 | 0.248 |
| sarcoma | 0.69 | 0.39-1.22 | 0.2 | 0.66 | 0.37-1.15 | 0.143 | 0.61 | 0.32-1.17 | 0.139 | 0.57 | 0.29-1.09 | 0.09 |
| **Age, years**  Referent: <30 | | | | | | | | | | | | |
| 30≤age<40 | 2.17 | 1.43-3.30 | 0.0003 | 1.86 | 1.22-2.83 | 0.004 | 2.59 | 1.67-4.02 | 0.00002 | 2.50 | 1.61-3.88 | 0.00004 |
| 40≤age<50 | 2.63 | 1.76-3.91 | 2x10^-6^ | 2.23 | 1.50-3.32 | 0.00008 | 4.70 | 3.14-7.03 | 6x10^-14^ | 4.24 | 2.83-6.34 | 2x10^-12^ |
| 50≤age<60 | 2.75 | 1.85-4.07 | 5x10^-7^ | 2.23 | 1.50-3.31 | 0.00007 | 5.80 | 3.90-8.63 | 3x10^-18^ | 5.10 | 3.43-7.58 | 8x10^-16^ |
| 60≤age<70 | 3.21 | 2.17-4.76 | 6x10^-9^ | 2.57 | 1.73-3.82 | 3x10^-6^ | 7.35 | 4.95-10.91 | 4x10^-23^ | 6.18 | 4.16-9.17 | 2x10^-19^ |
| 70≤age<80 | 4.14 | 2.80-6.14 | 1x10^-12^ | 3.22 | 2.17-4.77 | 6x10^-9^ | 9.21 | 6.21-13.66 | 3x10^-28^ | 7.25 | 4.88-10.76 | 8x10^-23^ |
| 80≤age | 5.73 | 3.86-8.50 | 4x10^-18^ | 4.16 | 2.80-6.19 | 2x10^-12^ | 14.67 | 9.88-21.79 | <1x10^-30^ | 10.69 | 7.19-15.89 | <1x10^-30^ |
| **Diagnosed year**  Referent: 2002-2007 | | | | | | | | | | | | |
| 2008-2013 | 0.97 | 0.93-1.01 | 0.095 | 1.04 | 1.00-1.08 | 0.054 | 0.91 | 0.87-0.96 | 0.0002 | 0.95 | 0.90-0.99 | 0.029 |
