# Supplementary material for: The incidence and survival of pancreatic cancer by histology, including rare subtypes: a nation‐wide cancer registry‐based study from Taiwan
Source: Cancer Med. 2018 Sep 27;7(11):5775–88. doi: 10.1002/cam4.1795 (PMC6246938; doi:10.1002/cam4.1795)
Supplement: Supplementary file 4 [file CAM4-7-5775-s004.docx]

**Supplementary Table 4.** One-year, three-year and five-year survival probability in pancreatic cancers by subtype in two time periods, 2002-2007 (T1) and 2008-2013 (T2)

|  | one year survival probability | | | three year survival probability | | | five year survival probability | | |
| --- | --- | --- | --- | --- | --- | --- | --- | --- | --- |
|  | All | Men | Women | All | Men | Women | ALL | Men | Women |
| **2002-2007 (T1)** |  |  |  |  |  |  |  |  |  |
| All | 0.2412 | 0.2355 | 0.249 | 0.0856 | 0.0825 | 0.0899 | 0.0604 | 0.0574 | 0.0645 |
| Adenocarcinoma | 0.270 | 0.2542 | 0.2931 | 0.075 | 0.0664 | 0.0861 | 0.049 | 0.0438 | 0.0559 |
| Carcinoma | 0.1792 | 0.1906 | 0.1631 | 0.0805 | 0.0874 | 0.0708 | 0.0617 | 0.0654 | 0.0565 |
| Neuroendocrine tumors | 0.619 | 0.641 | 0.6 | 0.381 | 0.359 | 0.4 | 0.238 | 0.1538 | 0.3111 |
| Endocrinomas | 0.755 | 0.72 | 0.7857 | 0.660 | 0.68 | 0.6429 | 0.528 | 0.56 | 0.5 |
| Lymphoma | 0.393 | 0.5 | 0.25 | 0.321 | 0.375 | 0.25 | 0.286 | 0.3125 | 0.25 |
| Squamous cell carcinoma | 0.182 | 0.25 | 0.1 | 0.046 | 0.0833 | - | 0.046 | 0.0833 | - |
| Small cell carcinoma | 0.125 | 0 | 0.25 | - | - | - | - | - | - |
| Sarcoma | 0.364 | 0.4286 | 0.25 | 0.273 | 0.2857 | 0.25 | 0.091 | - | 0.25 |
| **2008-2013 (T2)** |  |  |  |  |  |  |  |  |  |
| All | 0.2649 | 0.246 | 0.2899 | 0.097 | 0.0857 | 0.1117 | 0.0703 | 0.0615 | 0.082 |
| Adenocarcinoma | 0.302 | 0.2791 | 0.3342 | 0.085 | 0.071 | 0.1053 | 0.055 | 0.0414 | 0.0721 |
| Carcinoma | 0.1268 | 0.1239 | 0.1306 | 0.044 | 0.0486 | 0.0383 | 0.0347 | 0.0398 | 0.0284 |
| Neuroendocrine tumors | 0.736 | 0.7032 | 0.7696 | 0.599 | 0.5844 | 0.6132 | 0.506 | 0.5018 | 0.5095 |
| Endocrinomas | 0.784 | 0.7143 | 0.8333 | 0.705 | 0.665 | 0.7333 | 0.659 | 0.665 | 0.6561 |
| Lymphoma | 0.578 | 0.5556 | 0.6111 | 0.511 | 0.5185 | 0.5 | 0.511 | 0.5185 | 0.5 |
| Squamous cell carcinoma | 0.074 | 0.05 | 0.1429 | 0.037 | - | 0.1429 | 0.037 |  | 0.1429 |
| Small cell carcinoma | 0.120 | 0.15 | 0 | 0.040 | 0.05 | - | 0.040 | 0.05 | - |
| Sarcoma | 0.500 | 0.2857 | 0.7143 | 0.351 | 0.2857 | 0.4082 | 0.234 | 0.2857 | - |
